# Heterologous Secretory Expression and Characterization of Dimerized Bone Morphogenetic Protein 2 in* Bacillus subtilis*

**DOI:** 10.1155/2017/9350537

**Published:** 2017-11-23

**Authors:** Muhammad Umair Hanif, Roquyya Gul, Muhammad Imran Hanif, Abid Ali Hashmi

**Affiliations:** ^1^Institute of Molecular Biology and Biotechnology/Centre for Research in Molecular Medicine, The University of Lahore, 1-Km Defense Road, Bhubtian Chowk, Lahore, Pakistan; ^2^Gulab Devi Educational Complex, Gulab Devi Chest Hospital, Ferozpur Road, Lahore, Pakistan

## Abstract

Recombinant human Bone Morphogenetic Protein 2 (rhBMP2) has important applications in the spine fusion and ortho/maxillofacial surgeries. Here we first report the secretory expression of biological active dimerized rhBMP2 from* Bacillus subtilis* system. The mature domain of BMP2 gene was amplified from pTz57R/BMP2 plasmid. By using pHT43 expression vector two constructs, pHT43-BMP2-M (single BMP2 gene) and pHT43-BMP2-D (two BMP2 genes coupled with a linker to produce a dimer), were designed. After primary cloning (DH5*α* strain) and sequence analysis, constructs were transformed into* Bacillus subtilis* for secretory expression. Expression conditions like media (2xYT) and temperature (30°C) were optimized. Maximum 35% and 25% secretory expression of monomer (~13 kDa) and dimer (~25 kDa), respectively, were observed on SDS-PAGE in SCK6 strain. The expression and dimeric nature of rhBMP2 were confirmed by western blot and native PAGE analysis. For rhBMP2 purification, 200 ml culture supernatant was freeze dried to 10 ml and dialyzed (Tris-Cl, pH 8.5) and Fast Protein Liquid Chromatography (6 ml, Resource Q column) was performed. The rhBMP2 monomer and dimer were eluted at 0.9 M and 0.6 M NaCl, respectively. The alkaline phosphatase assay of rhBMP2 (0, 50, 100, 200, and 400 ng/ml) was analyzed on C2C12 cells and maximum 200 ng/ml activity was observed in dose dependent manner.

## 1. Introduction

Bone Morphogenetic Proteins (BMPs) are the members of the TGF-*β* superfamily and act as regulators during embryogenesis, bone, and cartilage repair [1]. Over 20 different members of the BMP subfamily had been identified and characterized to date in humans [2]. BMP binds through type I and type II serine/threonine kinase receptors and SMAD intracellular signaling pathway to regulate various biological processes [3]. Two members of this family, BMP-2 and BMP-7, had been approved by FDA as recombinant human proteins for clinical use and proved their significance in clinical orthopedic studies [1] and in oral/maxillofacial surgeries [4]. Generally BMPs are active in very low doses (5–20 ng/ml) but commercially available rhBMPs are used in very high doses (up to 40 mg) in some procedures [5]. Therefore, cost effect bulk production of rhBMP is mandatory for the therapeutic applications.

To date BMPs and other members of the TGF*β* family have been produced in various expression systems, mainly bacterial [6], yeast [7], and mammalian [8]. The mammalian expression system has the advantage of biologically active production of heterologous proteins but the manufacturing cost is high with low yield of protein recovery; also the system is difficult to scale up for the commercial production. In bacterial expression system,* E. coli* is the most widely used recombinant host but despite its cost effectiveness and ability to produce large quantities of heterologous proteins, it has a drawback that most proteins are produced in biologically inactive state with no posttranslational modifications [9]. Generally release of intracellular protein and* in vitro* refolding step is required to produce biological active proteins which increases the cost and reduces the protein recovery as protein is lost with additional processing. Other useful bacterial system is gram positive soil bacteria,* Bacillus subtilis (B. subtilis),* which has been known to secrete industrially relevant proteins directly into the culture media with no endo or exotoxins and has the status of GRAS (Generally Recognized as Safe) by US FDA [10]. Over the years many attempts are made and a large number of patents had been filed related to optimization of protein secretion by* B. subtilis* [11], although the secretion of homologous proteins from* B. subtilis* is straightforward with concentrations up to grams per liter [12], while the production of heterologous proteins is problematic and the optimization strategies for overexpression for one protein do not always work for the other proteins [11]. In short the optimization steps and protein quantities vary with the characteristics of the protein under study. Previously secretion of several homologous enzymes and industrial agents including nucleotides, vitamin riboflavin, and flavoring agents has been achieved [13, 14]. Regarding the heterologous secretion of proteins in different* Bacillus* species generally and especially in the class of cytokines BMP7, interleukin-2 (IL-2), IL-3, and Granulocyte Colony Stimulating Factor (GCSF) had successfully been produced in biologically active form with varying concentrations and purity [15–18] as shown in Table 1.

Considering the advantages of* Bacillus* expression system, the focus of the present study was to increase the yield of the secretory dimerized rhBMP2, its optimization, and characterization which has not been reported yet.

## 2. Materials and Methods

### 2.1. Bacterial Strains, Plasmids, and Cloning

*E. coli* strain; DH5*α* and* B. subtilis *strains; SCK6 and WB600 were used for cloning, subcloning, and rhBMP2 production, respectively. T/A cloning kit (InsTAclone catalog number K1213) containing the pTz57R/T plasmid was purchased from Thermo Scientific. pHT43 (catalog number PBS002)* Bacillus* expression vector was purchased from MoBiTec GmbH Germany. All the restriction enzymes were purchased from Thermo Scientific unless otherwise specified.

Human BMP2 gene was amplified from pTz57R/BMP2 plasmid (previously constructed in lab) with forward (FBMP2-M) and reverse (RBMP2-M) primers containing restriction sites (*Bam*HI/*Xba*I) and His-tag sequence at 3′ terminal (Table 2). The amplified product was run on 1% agarose and the specific band was eluted and cloned in pTz57R/T vector and transformed into DH5*α* to construct BMP monomer (pTz57R/BMP2-monomer). For the construction of BMP2 homodimer covalently coupled glycine serine rich linker (glycine_3_ serine_1_)_3_ along with* Bgl*II restriction site was incorporated. Human homodimer BMP2 gene was amplified with two different sets of primers (FBMP2-D/RBMP2-L and FBMP2-L/RBMP2-D) to generate two monomers as described above containing the restriction sites, linker sequence, and the His-tag at the 3′ end of the gene as shown in Table 2. Both the monomers of BMP2 were run on 1% agarose gel and specific bands were eluted out, cloned in pTz57R/T, and transformed into DH5*α*. To produce homodimer, both the monomers were first amplified, T/A cloned, and digested with* Bgl*II. Further, the two digested monomers were ligated by using T4 ligase and then amplified with FBMP2-D and RBMP2-D primers. The product was run on 1% agarose gel, cloned into pTz57R/T (pTz57R/BMP2-Dimer) and transformed in DH5*α* as described for the BMP2 monomer. Covalently linked BMP2 homodimer was referred to as BMP2 dimer and the single gene was referred to as BMP2 monomer in this work hereafter. Both constructs (pTz57R/BMP2-monomer and pTz57R/BMP2-dimer) were sequence analyzed for the confirmation of the sequence. The positive transformants were restricted with* Bam*HI/*Xba*I and ligated into properly restricted pHT43* Bacillus* expression vector. The plasmids for rhBMP2 production (pHT43-BMP2-M and pHT43-BMP2-D) were transformed into* B. subtilis* SCK6 and WB600 strains as essentially described earlier [22, 23].

### 2.2. Expression and Optimization of rhBMP2

A single positive transformant of each pHT43-BMP2-M and pHT43-BMP2-D plasmid was inoculated into 5 ml LB medium (5 g/L yeast extract, 10 g/L tryptone and 10 g/L NaCl) containing 5 *μ*g/ml chloramphenicol and incubated at 37°C, 200 rpm for overnight. Next morning, 25 ml LB medium was refreshed with 2% overnight culture and incubated under the conditions mentioned above until the OD600 reached 0.6–0.8. IPTG (1 mM) was induced to obtain the expression of the rhBMP2 monomer and dimer. A postinduction sample was taken at 2, 4, and 6 hours and cells were harvested by centrifugation at 13000 rpm for 5 min at 4°C. The culture supernatant was processed further for the confirmation of secretory protein expression with trichloroacetic acid (TCA) precipitation. Briefly 0.15% sodium deoxycholate was added in the culture medium and incubated at room temperature for 15 min. After that 150 *μ*l of 100% TCA solution was added and incubated for 10 min on ice. The sample was then centrifuged at 14000 rpm at 4°C for 10 min. The supernatant was discarded and the pellet was washed with 1 ml of 100% acetone solution twice and centrifuged at 14000 rpm for 10 min at 4°C during washing. The pellet was resuspended in 100 *μ*l of Tris-Cl buffer (pH 8.0) and run on 18% SDS-PAGE for expression analysis. After confirmation, the maximum expression of rhBMP2 monomer protein was optimized in three different media [LB, 2xYT (16 g/L tryptone, 5 g/L NaCl, 10 g/L yeast extract) and 2xL MAL (2% tryptone, 1% yeast extract, 1% NaCl, 7.5% maltose hydrate, and 7.5 *μ*g/mL MnSO4)] and at two different temperatures, that is, 30°C and 37°C in SCK6 and WB600 strains. The optimization of rhBMP2 monomer was done initially and then the dimer was grown on the preoptimized conditions of media, temperature, and the strain. Though, monomer and dimer were optimized individually in case of time span, IPTG, and lactose concentration.

### 2.3. Western Blot, Native PAGE, and Purification of rhBMP2

For western blot analysis, protein samples were run on 18% SDS-PAGE. After running of the gel, proteins were blotted on to presoaked nitrocellulose membrane by electroblotting using the transfer buffer [25 mM Tris, 192 mM glycine, and 6.20% v/v methanol (pH 8.3)] and run on 400 mA for 2.5 hours. Membrane was presoaked with 1x PBST [NaCl 3.0 gm, KH_2_PO_4_ 0.2 gm, NaHPO4 1.15 gm, and KCl 0.2 gm/L, (pH 7.4) containing 0.5% Tween-20] and blocking was done with blocking buffer [5% skimmed milk powder in 1x PBS (NaCl 3.0 gm, KH_2_PO_4_ 0.2 gm, NaHPO4 1.15 gm, and KCl 0.2 gm/L, pH 7.4)] and incubated for overnight at 4°C. The blocked membrane was washed with 1x PBS three times for 15 min each. Anti-human BMP2 antibody (Sino Biological catalog number 10426-MM03) at dilution of 1 : 500 specific to target protein was incubated with the membrane at room temperature for 2 hours. After washing of unbound primary antibody with 1x PBST the secondary antibody (goat polyclonal Ab to mouse IgG HRP; lot number GR129315-1) at 1 : 1000 dilution was added at room temperature for 2 hours. Protein was detected by 3,3′,5,5′ tetramethylbenzidine (TMB) substrate (Invitrogen), and reaction was stopped by 1 M H_2_SO_4._ Moreover, to confirm the dimeric form of the rhBMP2 protein 12% native PAGE was performed in nonreducing condition (without SDS and *β*-mercaptoethanol). The samples were neither boiled nor was SDS added in running buffer to maintain the native confirmation and structure of the protein.

The purification of rhBMP2 monomer and dimer proteins were performed in three steps. First a 200 ml of the culture supernatant was freeze dried to a final volume of 10 ml and then dialyzed against the Tris-Cl (pH 8.5). Then the proteins were purified using Hi-Trap 6 ml Resource Q column (GE Healthcare) using FPLC system (AKTA purifier GE Healthcare systems). A concentration gradient of NaCl was used to phase separate the proteins on the basis of ionic strength.

### 2.4. Biological Activity of rhBMP2

The biological activity of the rhBMP2 monomer and dimer proteins were determined on C2C12 cells by alkaline phosphatase (ALP) assay (catalog number ab83369 Abcam)* in vitro*. The cells were grown in complete Dulbecco's Modified Eagle Medium (DMEM) supplemented with 100 *μ*g/ml penicillin, 100 U/ml streptomycin, and 10% Fetal Bovine Serum (FBS) at 37°C and 5% CO_2_. 1× 10^5^ cells were seeded in the six-well plate and on reaching ~80% confluency, 24 hours prior to experiment, and cells were serum starved with DMEM medium containing 2% FBS. Purified rhBMP2 monomer and dimer were induced at 0, 50, 100, 200, and 400 ng/ml in DMEM medium without FBS. Commercially available rhBMP2 (SinoBiological catalog number 10426-HNAE-20) was used at 100 and 500 ng/ml dose as a positive control and DMEM was used as a negative control. After 72 hours of induction, the cells were washed with 1x PBS and processed for ALP assay. Briefly, the cells were lysed with the assay buffer and the cell lysate was used for the ALP estimation. A standard curve was generated of* p*NPP (0, 4, 8, 12, 16, and 20 nmols) substrate of ALP. The quantities of ALP in the test samples were calculated from the standard curve.

## 3. Results

### 3.1. Construction of BMP2 Monomer and Dimer

**Figure 1:**
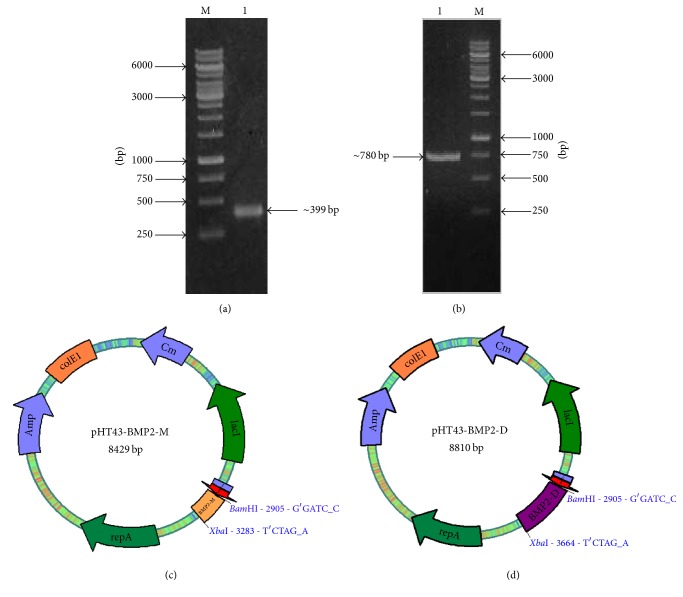
Amplification of BMP2 monomer and dimer and construction of recombinant plasmids. (a) Amplified PCR product of hBMP2 resolved on 1% agarose gel showing band of BMP2 monomer at ~399 bp. Lane M, 1 kb DNA ladder; Lane 1, hBMP2 monomer. (b) Amplified PCR product of hBMP2 dimer showing covalently linked BMP2 dimer at ~780 bp. Lane M, 1 kb DNA ladder; Lane 1, hBMP2 dimer. (c) Recombinant plasmid pHT43-BMP2-M (8429 bp) showing position of the hBMP2 monomer along with restriction sites. (d) Recombinant plasmid pHT43-BMP2-D (8810 bp) showing position of hBMP2 dimer along with restriction sites.

The BMP2 monomer was amplified from the already cloned plasmid (pTz57R/BMP2), which encodes the mature peptide of BMP2 that constitutes ~399 bp [Figure 1(a)]. For the construction of the BMP2 homodimer, BMP2 gene was amplified with two different sets of primers containing a linker sequence. The two monomers were digested and ligated within the linker region by using T4 DNA ligase and amplified to generate homodimer of ~780 bp [Figure 1(b)]. The purified amplicons of BMP2 monomer and dimer were T/A cloned using pTz57R/T vector. Both the monomer and dimer were confirmed with the restriction analysis and further verified and screened for possible mutations by sequencing. The restricted (*Bam*HI/*Xba*I) and purified BMP2 monomer and dimers were subcloned into the pHT43 vector for the expression study in* B. subtilis* as shown in Figures 1(c) and 1(d). The secondary cloning was confirmed by the colony PCR and restriction analysis (data not shown).

### 3.2. Secretory Expression of rhBMP2 in* B. subtilis*

**Figure 2:**
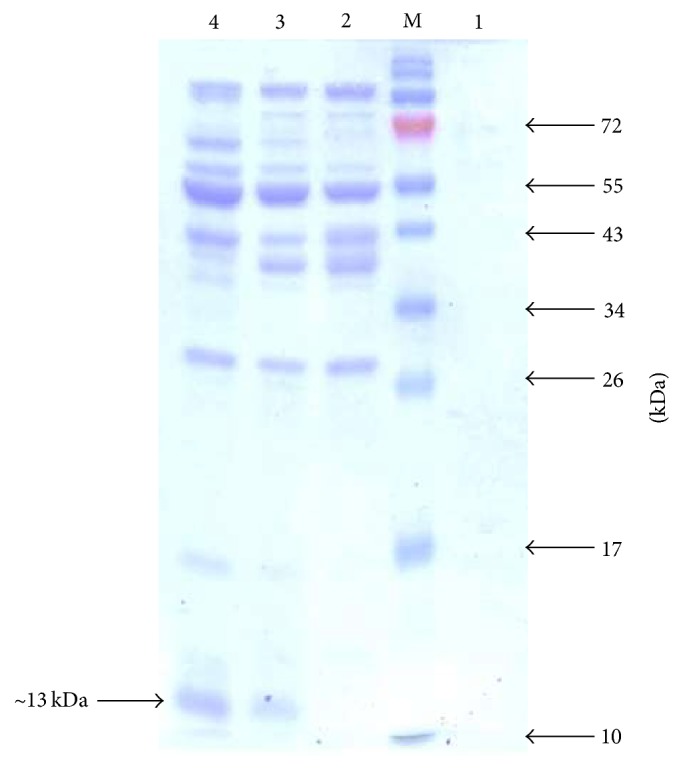
Expression of rhBMP2 monomer in 2xYT medium at 37°C showing band at ~13 kDa. Lane M, protein marker; Lane 1, uninduced sample; Lanes 2–4, showing 2, 4, and 6 hours' postinduction samples.

**Figure 3:**
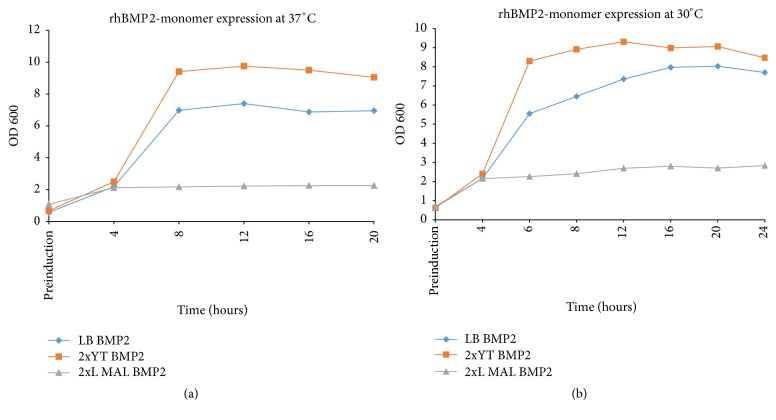
Graphical representation of growth curve of rhBMP2 monomer at 37°C and 30°C in different media and at different time points. *x*-axis showing the time scale up to 24 hours and *y*-axis showing the absorbance at 600 nm. (a) Growth curve at 37°C and maximum growth was observed in 2xYT medium. (b) Growth curve at 30°C in LB, 2xYT, and 2xL MAL media.

**Figure 4:**
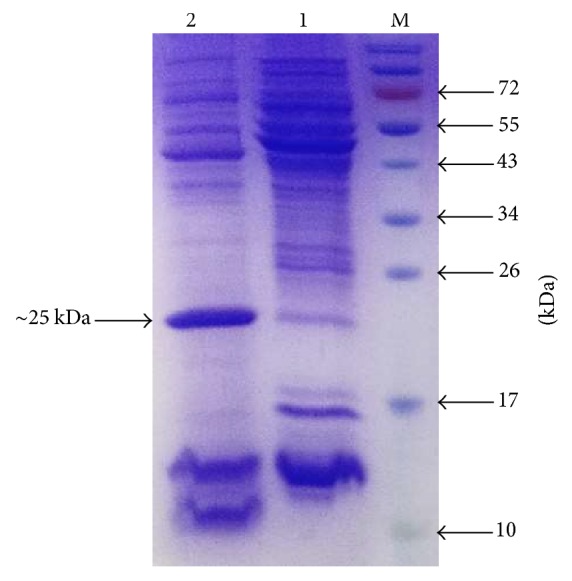
Expression of rhBMP2 dimer in 2xYT medium at 30°C. Lane M, protein marker; Lane 1, uninduced sample; Lane 2, rhBMP2 dimer induced with IPTG showing band at ~25 kDa.

The initial secretory expression of rhBMP2, using expression vector pHT43-BMP2-M, was confirmed at 37°C in 2xYT medium in SCK6 strain as shown in Figure 2. The rhBMP2 monomer expression was optimized in both strains (SCK6 and WB600) using three different media and at two temperatures, that is, 30°C and 37°C, as mentioned in Materials and Methods. According to the results shown in Figures 3(a) and 3(b) the maximum growth was observed at 37°C in 2xYT media and SCK6 strain as determined by OD600 values, whereas the maximum secretory expression of rhBMP2 monomer (~35%) was observed at 30°C after 20 hours of fermentation (see Supplementary Figures S1 and S2 in Supplementary Material available online at https://doi.org/10.1155/2017/9350537). However in case of WB600 strain, secretory expression (~45%) was observed at only 37°C (Supplementary Figure S3) and a very low level of expression was observed at 30°C after 24 hours (Supplementary Figure S4) in 2xYT medium, while no expression was visible in LB and 2xL MAL medium (data not shown). The WB600 yielded higher secretory expression at 37°C than SCK6 at 30°C in 2xYT medium but it was not proceeded further due to very high frequency of plasmid instability and early loss of expression. However, to obtain the maximum expression of rhBMP2 dimer, the already optimized conditions in case of rhBMP2 monomer like temperature and media were used. Maximum 25% of secretory expression of rhBMP2 dimer was achieved at 30°C in 2xYT medium in SCK6 strain after 8 hours of fermentation (Figure 4). IPTG concentration of 0.6 mM was optimized to attain the maximum expression of both rhBMP2 monomer and dimer. However in case of lactose induced expression, rhBMP2 monomer and dimer yielded maximum expression at 6 mM and 10 mM, respectively. The fermentation time for rhBMP2 monomer and dimer was optimized at 24 and 16 hours, respectively, by utilizing 10 mM of lactose (data not shown).

### 3.3. Characterization of rhBMP2

**Figure 5:**
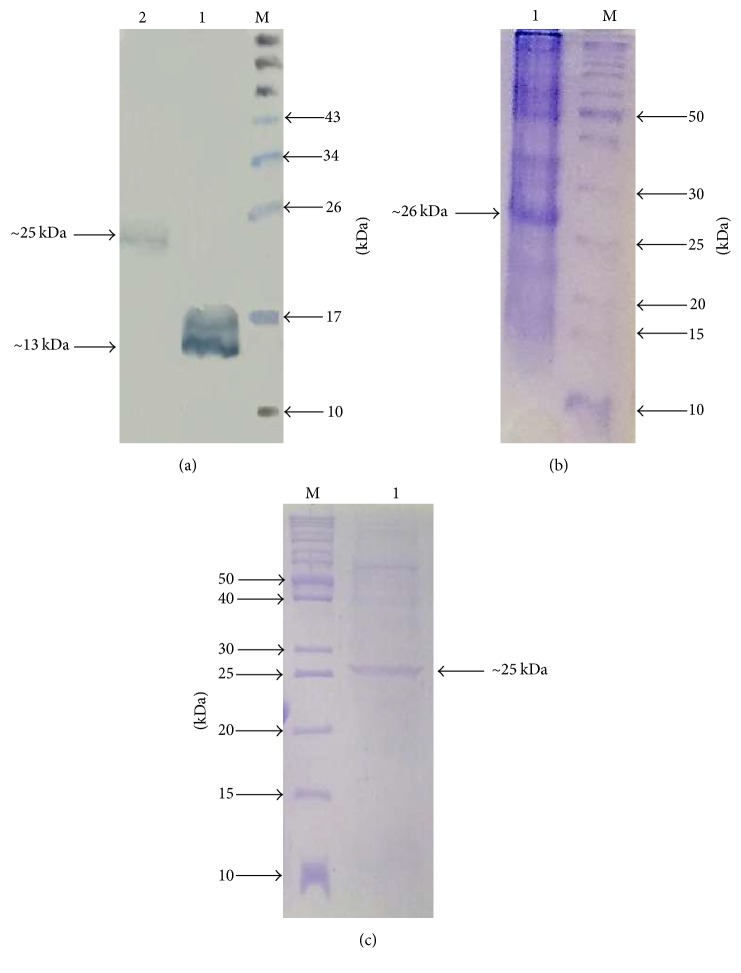
Characterization of rhBMP2 monomer and dimer. (a) Western blot analysis of rhBMP2 monomer and dimer. Lane M, protein marker; Lane 1, rhBMP2 monomer showing band at ~13 kDa; Lane 2, rhBMP2 dimer showing band at ~25 kDa. (b) Native PAGE analysis of rhBMP2 monomer showing dimerized form. Lane M, protein marker; Lane 1, nonreducing rhBMP2 monomer showing band at ~26 kDa. The band is slightly above the 26 kDa marker band due to nonreducing nature of the sample. (c) 18% SDS-PAGE showing band of rhBMP2 dimer at ~25 kDa after collecting supernatant from 50 kDa Amicon Ultra centrifugal column.

The expression of rhBMP2 monomer and dimer was further confirmed by the western blot analysis. Antibody against the rhBMP2 protein showed two distinct bands at ~13 kDa and ~25 kDa for monomer and dimer, respectively [Figure 5(a)]. The dimeric nature of the BMP2 monomer was confirmed by the native PAGE analysis and showed a distinct band at ~26 kDa [Figure 5(b)]. We did not perform the native PAGE analysis of covalently linked dimer of rhBMP2 due to its size similarity with disulfide linked dimer of BMP2. We hypothesize that the covalently linked dimer of BMP2 is producing dimer of the dimer as it has two cysteine residues available for the disulfide bond formation. This was confirmed by size exclusion chromatography based centrifugal column (Amicon Ultra-15 Centrifugal Filter Units Millipore catalog number UFC905024) with a cut-off value of 50 kDa. It showed that all the protein was retained and concentrated on the membrane of the column and nothing was retrieved in the flow through the column which suggested the correctness of our hypothesis [Figure 5(c)].

rhBMP2 monomer and dimer were first purified by using Nickel column due to the incorporation of His-tag at 3′ end of each protein. As a result, all of the rhBMP monomer and dimer were eluted in the flow through. This showed that the His-tag at 3′ end was being masked in both cases of rhBMP monomer and dimer (Supplementary Figure S5). Further to sort out the problem, purification was done by ion exchange chromatography using Hi-Trap Resource Q column (6 ml) using an AKTA Purifier FPLC system. The peaks shown in the chromatogram of the monomer and dimer corresponds to the concentration gradient of 0.9 M and 0.6 M NaCl solution and the elution volume (Supplementary Figure S6). The fractions were pooled and run on 15% SDS-PAGE. Approximately 90% and 80% purity were achieved in case of rhBMP2 monomer and dimer, respectively. Final percentage recovery of 1.12% and 3.17% for rhBMP2 monomer and dimer, respectively, was achieved (Table 3).

**Figure 6:**
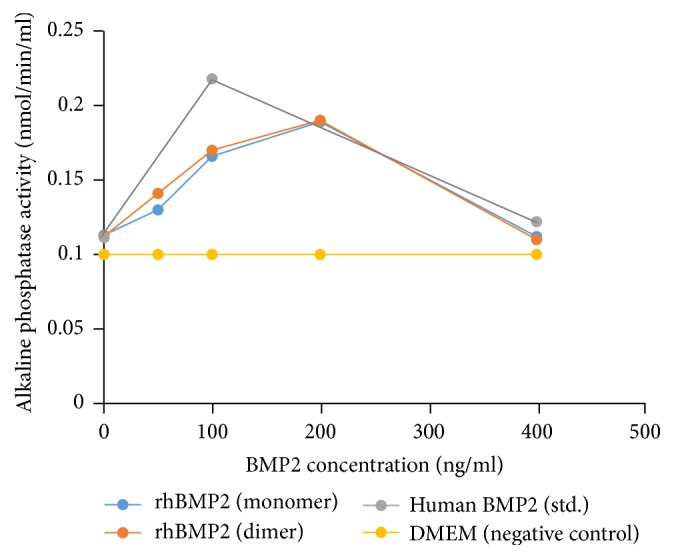
Biological activity of rhBMP2 monomer and dimer estimated by ALP assay and measured at nmol/ml/min. (a) Standard curve of ALP. *x*-axis showing the* p*NPP conc. (nmol) and *y*-axis showing the absorbance at 405 nm. (b) Different concentrations of rhBMP2 monomer and dimer (0, 50, 100, 200, and 400 ng/ml) were tested and maximum activity was observed at 200 ng/ml, respectively, in dose dependent manner. Higher dose (400 ng/ml) showed reduce activity. Positive control (human BMP standard) was tested at 100 and 500 ng/ml doses and showed reduce activity at 500 ng/ml. Negative control (DMEM) showed negligible ALP activity.

The rhBMP2 monomer and dimer were further characterized by their biological activity. The induction of BMP2 on myoblastic and osteoblastic cell lines was reported to stimulate the ALP expression. ALP produced in this reaction was used to hydrolyze the known amount of* p*NPP which acts as the substrate of ALP. Thus the amount of* p*NPP hydrolyzed is directly proportional to the amount of ALP produced by the induction of BMP2. Our results showed that the ALP levels were raised in the dose dependent manner of rhBMP2 monomer and dimer as shown in Figure 6. It was observed that at 200 ng/ml of rhBMP2 monomer and dimer had the maximum activity on C2C12 cells* in vitro*, whereas the higher dose (>400 ng/ml) seems to have an inhibitory effect as evidenced by the decline in the ALP activity which was further confirmed by the commercially available rhBMP2 positive control as well. However, the negative control, that is, DMEM, showed negligible ALP activity.

## 4. Discussion

The* Bacillus* secretory expression system was successfully employed for the extracellular secretion of rhBMP2 in dimeric biologically active form. A major limitation of this system is the production of extracellular proteases which degrades the recombinant foreign proteins [24]. In this study two strains were used SCK6 which is genetically modified and has higher competency to uptake foreign DNA molecules and has upregulated expression of ComK gene under the xylose inducible promoter [22]. The other strain was WB600 which is deficient in six extracellular proteases and suitable for the extracellular production of the recombinant proteins [25]. In the current study up to 1 mg and 1.8 mg per 200 ml culture of rhBMP2 monomer and dimer, respectively, were produced successfully.

The BMP2 gene was successfully cloned in monomer and dimeric form into* B. subtilis* strains SCK6 and WB600. Expression vectors pHT43-BMP2-M and pHT43-BMP2-D contained the Pgrac strong promoter and signal peptide sequence of amyQ (*α* amylase) gene of* Bacillus amyloliquefaciens *which has been reported for the efficient secretion of recombinant proteins through Sec pathway [26]. The final destination of recombinant proteins depends on the presence or absence of signal peptide which serves as the global positioning system; any protein produced without signal peptide is retained in the cytoplasm [27]. In Sec dependent secretory pathway, proteins produced as preprotein complex with an N-terminal signal peptide translocate to the cell membrane where it binds to the secretory translocase complex recognized by the signal peptide. Protein is transported out of the cell after the removal of signal peptide at the cleavage site by specific signal peptidase [28, 29]. Thus, the selection of suitable signal peptide affects the rate of protein secretion and ultimately the yield of the secreted protein. One of the choices to select the signal peptide is the usage of commercially available signal peptides, literature survey, and review of proteome of the host organism for signal peptide. The latter is linked to the high yield of the homologous secreted proteins [11, 27]. In this study we have used the signal peptide of *α*-amylase which has been reported for high secretion of homologous protein and have well defined cleavage site. Moreover, its usage is also reported for heterologous proteins, for example, extracellular secretion of human proteins including IL-3 and interferon *α*2 (INF*α*2) [16, 20].

Generally bacterial expression systems are unable to produce proteins in dimeric form and it is reported previously that rhBMP2 produced from* E. coli* was in an inactive and monomeric form [9, 30]. To overcome this problem a covalently linked homodimer of rhBMP2 consisting of glycine serine rich linker between two monomers of BMP2 was generated. This strategy had been reported previously in the case of heterodimers BMP2/BMP7 and some other proteins of BMP family and proved to be useful in the generation of active heterodimers [31]. In this study we have successfully generated the active form of homodimer with total 25% of the secretory expression. However, the maximum secretory expression of the monomer was obtained to be 35%. Interestingly, WB600 cells showed high protein expression at 37°C as compared to SCK6 but with the limitation of plasmid instability. Previous data reports that plasmid instability is not a rare phenomenon in* B. subtilis* [11, 24]. The plasmid instability is either segregational or structural; the segregational instability is more common in plasmids with low copy number, while the structural instability is difficult to detect with conventional selection pressure [32]. However, we did not face any such problem in SCK6 strain. Extracellular proteases are another major limitation of the* Bacillus* secretory expression system and among all the* Bacillus* strains, WB600 is deficient in six extracellular proteases which makes it suitable host for the production of secretory proteins. However, these protease deficient strains are more prone to cell lysis and ultimately reduce the growth which in turn leads to plasmid instability [33]. In our study the loss of expression in WB600 could also be due to secretion stress as the vector used in this study was of high copy number and large amount of protein accumulation in the pseudo (periplasmic) membrane can often lead to reduced growth and even cell death [28]. The hypothesis of secretion stress as the cause of expression loss was established on the observation that plasmid pHT43 contained* Col*E1 origin of replication which is associated with relaxed type of replication control and high copy number [34]. Furthermore, the presence of repA protein ensures the theta mode of replication which is derived from the naturally isolated pTB19 and pAM*β*1 larger plasmids of* Bacillus* specie [26]. Moreover, repA replication region shares no sequence homology with the rolling circle replication regions which is highly conserved in smaller plasmids and does not lead to the accumulation of ssDNA [35, 36], thus preventing the structural instability of the plasmid. Further the high initial expression of rhBMP2 in WB600 even at low cell density strengthens our hypothesis of secretion stress due to high copy number and greater metabolic stress. Although this is beyond the context of the current study to rule out the exact cause, it opens the door for the future.

Generally the yield of the secreted proteins is less as compared to the intracellular proteins.* B. subtilis* has been reported to secrete large quantities of enzymes into the culture medium mainly the extracellular proteases. Previous studies report that the homologous industrial enzymes like proteases were produced up to grams/L by* B. subtilis* [11, 23]. But the production and secretion of heterologous proteins are not that straightforward. Previous studies on recombinant production of human proteins from* B. subtilis* reported varying levels of secretory yield from as low as 100 *μ*g in case of IL-3 to 1000 mg in case of proinsulin. It is important to note that all those studies (Table 1) deployed different sets of conditions including vectors used with different signal peptide and different composition of media [16–21]. Specifically to BMP family, only BMP7 had been produced in* B. subtilis* other than rhBMP2 which was produced in the current study. The difference resides among the signal peptide, vector, and medium used; pLip signal peptide under the control of HpaII promoter was used in BMP7 production in LBL (Luria Broth + Lactose) medium [15] whereas we have used amyQ signal sequence under Pgrac promoter control with 2x YT medium. At least, in context of BMP family we have shown that our optimized conditions provided improved extracellular production of the rhBMP2. Other proteins including IL-3, IL-2, GCSF, Epidermal Growth factor (EGF), INF*α*2, and proinsulin had been produced with different set of conditions but the overall yield is considerably low as compared to* E. coli* system [15–18]. Previously Westers et al. have tested different sets of promoters and signal peptides along with three different types of media for IL-3 production [16]. They have reported a gross increase in yield from 100 *μ*g to 100 mg/L by changing the combination of promoter and signal peptide. According to them the best combination was the use of *α* amylase signal peptide with pHT43 promoter, which resulted in the maximum secretion of IL-3 protein and near zero cytoplasmic retention. These findings had also confirmed our use of amyQ signal peptide for efficient secretion. Although they also have encountered the loss of expression in protease deficient strain (WB700), in this study we achieved 5 mg/L in case of rhBMP2 monomer and 9 mg/L in case of rhBMP2 dimer with the percentage purity of 90% and 80%, respectively. However, Kim et al. reported that BMP7 was produced in the concentration of 0.8 *μ*g from a 3 L culture with the recovery of 58.5% and purity of 57.1%, respectively. We have achieved the final yield and purity greater than the BMP7 by Kim et al. [15]. The low yield of the heterologous proteins makes it difficult to purify with sufficient high recovery. Generally multiple steps are required to achieve a considerable amount of percentage purity. First the proteins have to be concentrated in the culture supernatant. We have freeze dried the proteins to reduce the volume of the culture supernatant to make it sufficiently low for the FPLC purification system. Then the protein was dialyzed through a suitable size of dialysis membrane (6–8 kDa) for the exchange of buffer and followed by a column purification step and at the end an additional step of size exclusion or gel filtration to achieve the maximum purity.

Previously in case of the BMP7, IL-2, IL-3, EGF, INF*α*2, proinsulin, and GCSF as well as in our study all the proteins produced by the* Bacillus* expression system were biologically active and in proper refolded configuration. This might be due to the presence of the molecular chaperons which aid in the refolding of the proteins and presence of the oxidoreductases which help in disulfide bond formation and dimer generation [11, 33]. Although it has an advantage of proper refolding and soluble and dimeric protein production the labor intensive purification process and the low yields make it unsuitable expression system for the production of mammalian proteins and especially of the BMP family.

## 5. Conclusions

In conclusion, we have successfully produced dimerized bioactive rhBMP2 as secretory protein from* B. subtilis* system. Furthermore, the covalently linked homodimer of rhBMP2 was also produced in tetramer conformation along with the dimeric configuration. Further improvement in terms of protein yield and secretion can be done by optimizing media and the combination of signal peptide along with promoter. But special focus is required in selecting the vector as very high copy number of the vector can lead to secretion stress which ultimately leads to expression loss. Also for the heterologous proteins, the optimization strategy and the expression level varies dramatically from protein to protein and cannot be generalized.

## Supplementary Material

Figure S1: Time span of rhBMP2 monomer at 37°C in 2xYT medium in SCK6 strain. Lane M, Protein marker; Lane 1, Pre-induction; Lane 2-7, 4, 8, 12, 16, 20 and 24 hours post induction samples respectively. Maximum expression shown at 12 hours of fermentation.
Figure S2: Time span of rhBMP2 monomer at 30°C in 2xYT medium in SCK6 strain. Lane M, Protein marker. Lane 1, Pre-induction; Lane 2-8, 4, 6, 8, 12, 16, 20, 24 hours post induction respectively. Maximum secretory expression (~35%) shown at 20 hours of fermentation.
Figure S3: Time span of rhBMP2 monomer in 2xYT medium at 37°C in WB600 strain. Lane M, Protein marker. Lane 1, Pre-induction; Lane 2-8, 4, 6, 8, 12, 16, 20 and 24 hours post induction samples. Maximum expression (~45%) observed at 24 hours of fermentation.
Figure S4: Time span of rhBMP2 monomer in 2xYT medium at 30°C in WB600 strain. Lane M, Protein marker; Lane 1, Pre-induction; Lane 2-8, 4, 6, 8, 12, 16, 20 and 24 hours post induction samples. Maximum expression observed only at 24 hours of fermentation.
Figure S5: Computational model of rhBMP2 dimer. The rhBMP2 monomer with His-tag at C-terminal was generated by I-Tasser web server () and protein-protein interaction was predicted by Haddock web server (). Model shows the C-terminal at His-tag is buried in the interior of the protein. Due to masking of His-tag, rhBMP2 was not purified by Nickel-NTA affinity chromatography.
Figure S6: Purification of rhBMP2 monomer and dimer by anion-exchange chromatography. (a) Chromatogram of rhBMP2 monomer showing peak at ~0.9M NaCl concentration. X-axis showing the volume of fractions (ml) and Y-axis showing the absorbance at 280nm. (b) Purified rhBMP2 monomer was run on 18% SDS-PAGE showing ~90% purity. Lane M, Protein marker; Lane 1, purified rhBMP2 monomer. (c) Chromatogram of rhBMP2 dimer showing peak at ~0.6M NaCl concentration. (d) Purified rhBMP2 dimer run on 18% SDS-PAGE showing ~80% purity. Lane M, Protein marker; Lane 1, purified rhBMP2 dimer.

## Figures and Tables

**Table 1:** Comparison of different recombinant human proteins produced in *Bacillus *species.

Recombinant human proteins	Vector	Media	Yield	Species	Reference
Epidermal Growth Factor	pM2Veg	LB	7.0 mg/L	*B. subtilis*	[19]
Interferon-*α*2	pKTH68	Broth-starch Medium	0.5–1.0 mg/L	*B. subtilis*	[20]
Interleukin 3	pMA5 derivatives	2X YT, MXR, MSR	100 *μ*g–100 mg/L	*B. subtilis*	[16]
Granulocyte Colony Stimulating Factor	pNWPH	2X L-MAL	96 mg/L	*B. subtilis*	[18]
Proinsulin	pSubP14	Mineral medium (MMG)	1000 mg/L	*B. subtilis*	[21]
BMP7	pBPT62	LBL	350 ng/L	*B. subtilis*	[15]
Interleukin 2	pNU211	3PY	120 mg/L	*B. brevis*	[17]
**rhBMP2**	**pHT43**	**2X YT**	**5–9 mg/L**	***B. subtilis***	**Current study**

**Table 2:** Sequence of hBMP2 monomer and dimer primers showing nucleotide and amplicon size (bp). The bold and underline sequences show restriction sites and His-tag (3′), respectively, incorporated in the primers.

Set of primers	Sequence (5′-3′)	Nucleotide (bp)	Amplicon size (bp)
FBMP2-M	CTATGCTTAGCAGGG**GGATCC**CAAGCCAAACACAAAC	37	399
	*Bam*HI		
RBMP2-M	**TCTAGA**TTAATGGTGGTGGTGATGATGACCGCGACCGCGACACCCACAACCCTC	54	
	*Xba*I		
FBMP2-D	CTATGCTTAGCAGGG**GGATCC**	21	384
	*BamH*I		
RBMP-L	**AGATCT**ACTACCTGAACTACTGCGACACCCACAACCCTCC	40	
	*Bgl*II		
FBMP-L	**AGATCT**TCAGGTTCGTCTAGTGGTCAAGCCAAACACAAAC	40	402
	*Bgl*II		
RBMP2-D	**TCTAGA**TTAATGGTGGTGGTGATGATG	27	
	*Xba*I		

**Table 3:** Summary of percentage recovery of rhBMP2 monomer and dimer at different steps of production from *B. subtilis.*

Purification steps	rhBMP (monomer)				rhBMP (dimer)			
	Volume (ml)	Total protein (mg)^a^	Purity (%)^b^	Recovery (%)	Volume (ml)	Total protein (mg)^a^	Purity (%)^b^	Recovery (%)
Cultural supernatant	200	90	35	100	200	60	25	100
Concentrated by freeze drying	10	89	35	98	5	58	25	96
FPLC	12	1	90	1.12	7	1.84	80	3.17

^a^Protein concentration was determined by absorbance measurements at OD_280_. ^b^The  % age purity was determined by densitometric analysis of 15% SDS-gel.

## Abbreviations

ALP: Alkaline phosphatase
amyQ: *α*-Amylase
*B. subtilis*: * Bacillus subtilis*
BMP: Bone Morphogenetic Protein
DMEM: Dulbecco's Modified Eagle Medium
*E. coli*: * Escherichia coli*
FBS: Fetal Bovine Serum
FPLC: Fast Protein Liquid Chromatography
GRAS: Generally Recognized as Safe
GCSF: Granulocyte Colony Stimulating Factor
hBMP: human Bone Morphogenetic Protein
IPTG: Isopropyl *β*-D-1-thiogalactopyranoside
IL-2: Interleukin 2
IL-3: Interleukin 3
PAGE: Polyacrylamide Gel Electrophoresis
*p*NPP: * para*-Nitrophenylphosphate
rhBMP: Recombinant human Bone Morphogenetic Protein
SDS-PAGE: Sodium Dodecyl Sulfate Polyacrylamide Gel Electrophoresis
TCA: Trichloroacetic acid
TGF*β*: Transforming Growth Factor beta
TMB: 3,3′,5,5′-Tetramethylbenzidine.
