# Supplementary material for: Heterologous Secretory Expression and Characterization of Dimerized Bone Morphogenetic Protein 2 in Bacillus subtilis
Source: Biomed Res Int. 2017 Nov 23;2017:9350537. doi: 10.1155/2017/9350537 (PMC5733156; doi:10.1155/2017/9350537)
Supplement: Supplementary file 1 — Figure S1: Time span of rhBMP2 monomer at 37°C in 2xYT medium in SCK6 strain. Lane M, Protein marker; Lane 1, Pre-induction; Lane 2-7, 4, 8, 12, 16, 20 and 24 hours post induction samples respectively. Maximum expression shown at 12 hours of fermentation. Figure S2: Time span of rhBMP2 monomer at 30°C in 2xYT medium in SCK6 strain. Lane M, Protein marker. Lane 1, Pre-induction; Lane 2-8, 4, 6, 8, 12, 16, 20, 24 hours post induction respectively. Maximum secretory expression (~35%) shown at 20 hours of fermentation. Figure S3: Time span of rhBMP2 monomer in 2xYT medium at 37°C in WB600 strain. Lane M, Protein marker. Lane 1, Pre-induction; Lane 2-8, 4, 6, 8, 12, 16, 20 and 24 hours post induction samples. Maximum expression (~45%) observed at 24 hours of fermentation. Figure S4: Time span of rhBMP2 monomer in 2xYT medium at 30°C in WB600 strain. Lane M, Protein marker; Lane 1, Pre-induction; Lane 2-8, 4, 6, 8, 12, 16, 20 and 24 hours post induction samples. Maximum expression observed only at 24 hours of fermentation. Figure S5: Computational model of rhBMP2 dimer. The rhBMP2 monomer with His-tag at C-terminal was generated by I-Tasser web server () and protein-protein interaction was predicted by Haddock web server (). Model shows the C-terminal at His-tag is buried in the interior of the protein. Due to masking of His-tag, rhBMP2 was not purified by Nickel-NTA affinity chromatography. Figure S6: Purification of rhBMP2 monomer and dimer by anion-exchange chromatography. (a) Chromatogram of rhBMP2 monomer showing peak at ~0.9M NaCl concentration. X-axis showing the volume of fractions (ml) and Y-axis showing the absorbance at 280nm. (b) Purified rhBMP2 monomer was run on 18% SDS-PAGE showing ~90% purity. Lane M, Protein marker; Lane 1, purified rhBMP2 monomer. (c) Chromatogram of rhBMP2 dimer showing peak at ~0.6M NaCl concentration. (d) Purified rhBMP2 dimer run on 18% SDS-PAGE showing ~80% purity. Lane M, Protein marker; Lane 1, purified rhBMP2 dimer. [file 9350537.f1.pdf]

SUPPLEMENTARY FIGURES

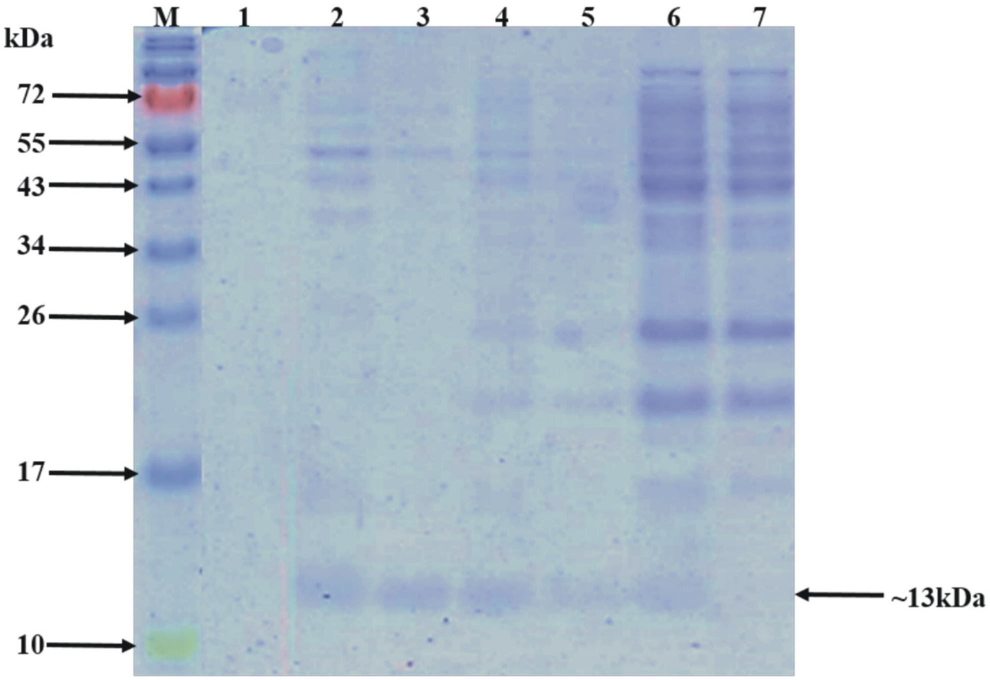

Figure S1: Time span of rhBMP2 monomer at 37°C in 2xYT medium in SCK6 strain. Lane M, Protein marker; Lane 1, Pre-induction; Lane 2-7, 4, 8, 12, 16, 20 and 24 hours post induction samples respectively. Maximum expression shown at 12 hours of fermentation.

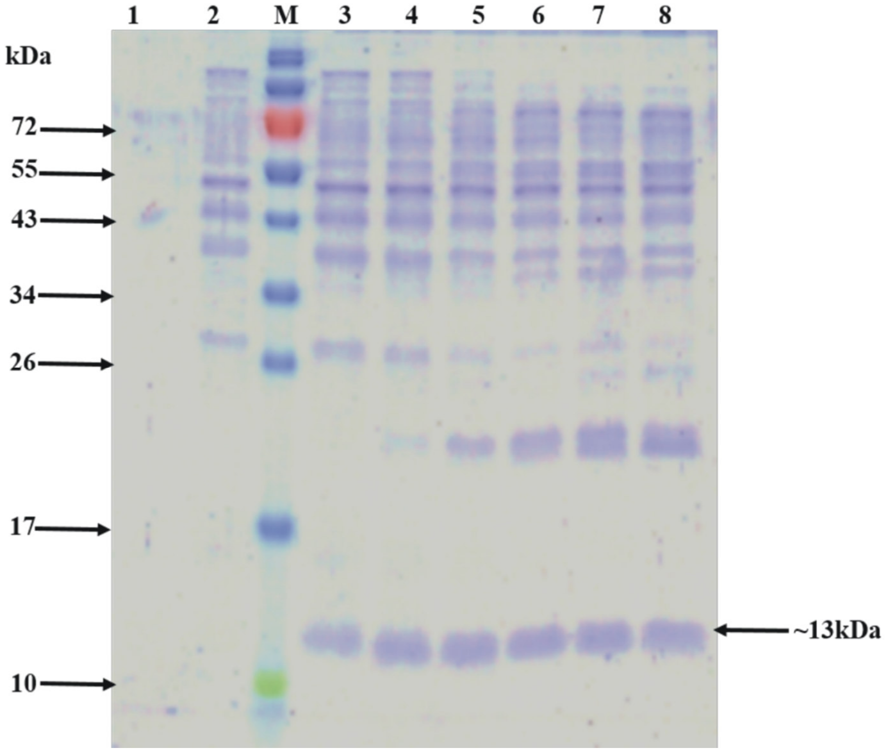

Figure S2: Time span of rhBMP2 monomer at 30°C in 2xYT medium in SCK6 strain. Lane M, Protein marker. Lane 1, Pre-induction; Lane 2-8, 4, 6, 8, 12, 16, 20, 24 hours post induction respectively. Maximum secretory expression (~35%) shown at 20 hours of fermentation.

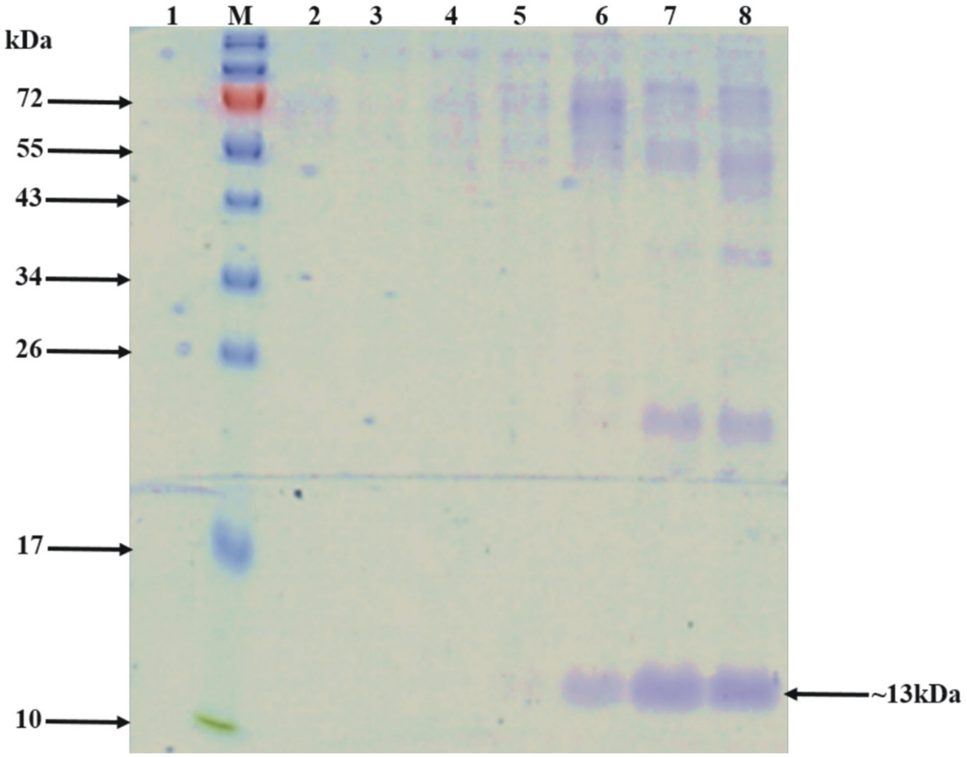

Figure S3: Time span of rhBMP2 monomer in 2xYT medium at 37°C in WB600 strain. Lane M, Protein marker. Lane 1, Pre-induction; Lane 2-8, 4, 6, 8, 12, 16, 20 and 24 hours post induction samples. Maximum expression (~45%) observed at 24 hours of fermentation.

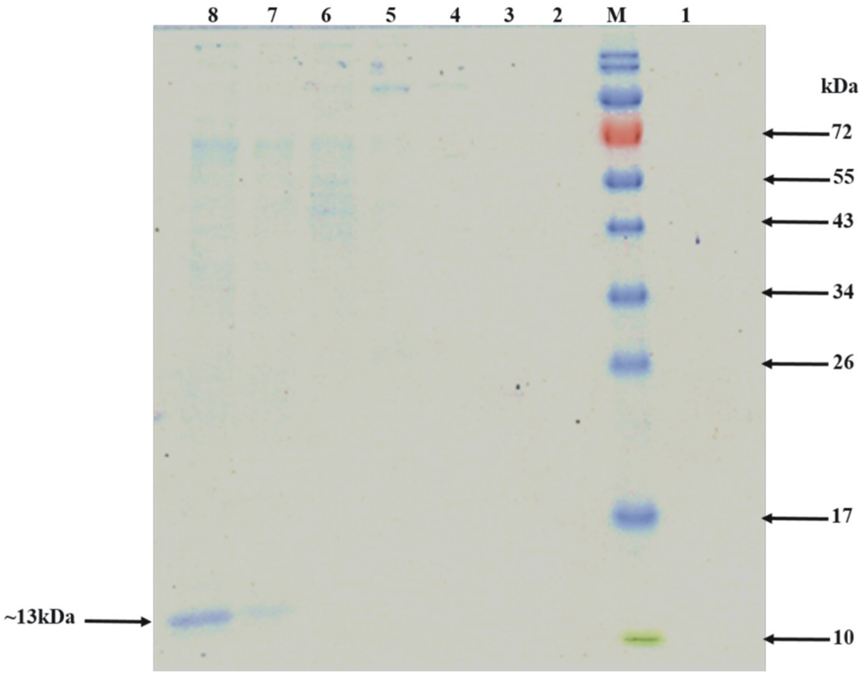

Figure S4: Time span of rhBMP2 monomer in 2xYT medium at 30°C in WB600 strain. Lane M, Protein marker; Lane 1, Pre-induction; Lane 2-8, 4, 6, 8, 12, 16, 20 and 24 hours post induction samples. Maximum expression observed only at 24 hours of fermentation.

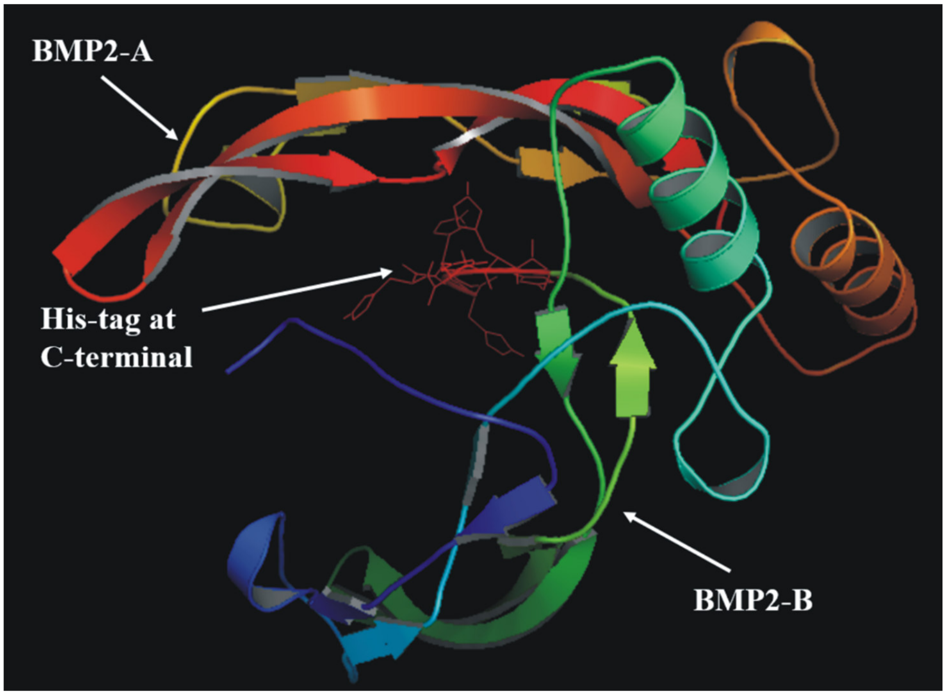

Figure S5: Computational model of rhBMP2 dimer. The rhBMP2 monomer with His-tag at C-terminal was generated by I-Tasser web server (https://zhanglab.ccmb.med.umich.edu/I-TASSER/) and protein-protein interaction was predicted by Haddock web server (http://haddock.science.uu.nl/services/HADDOCK/). Model shows the C-terminal at His-tag is buried in the interior of the protein. Due to masking of His-tag, rhBMP2 was not purified by Nickel-NTA affinity chromatography.

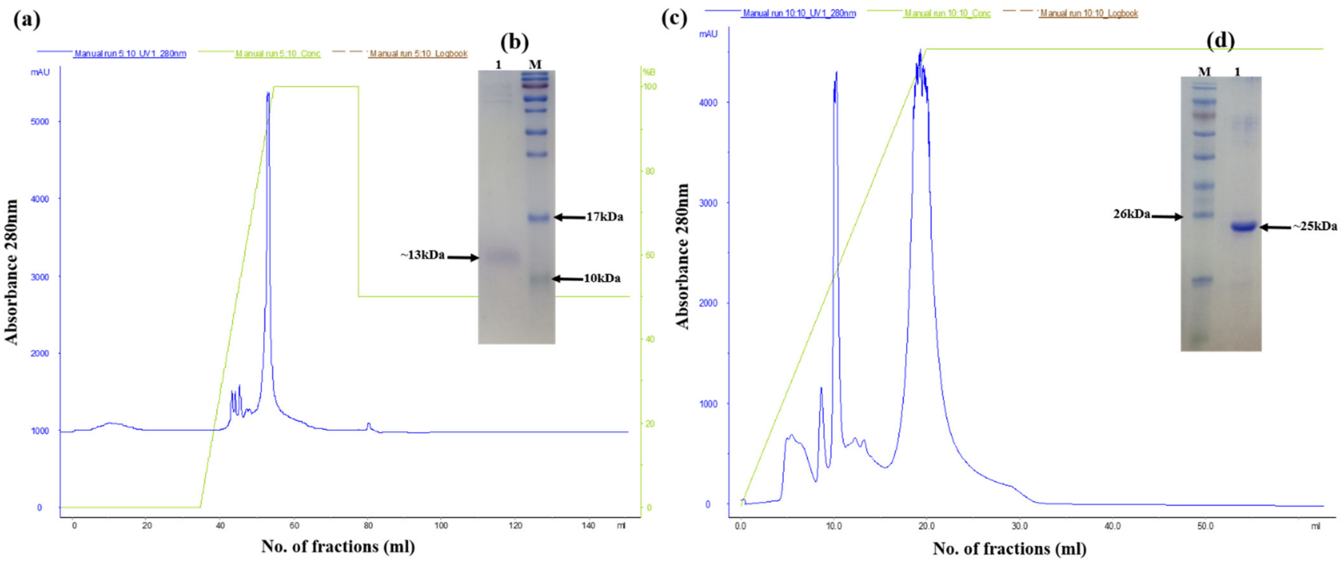

Figure S6: Purification of rhBMP2 monomer and dimer by anion-exchange chromatography. (a) Chromatogram of rhBMP2 monomer showing peak at ~0.9M NaCl concentration. X-axis showing the volume of fractions (ml) and Y-axis showing the absorbance at 280nm. (b) Purified rhBMP2 monomer was run on 18% SDS-PAGE showing ~90% purity. Lane M, Protein marker; Lane 1, purified rhBMP2 monomer. (c) Chromatogram of rhBMP2 dimer showing peak at ~0.6M NaCl concentration. (d) Purified rhBMP2 dimer run on 18% SDS-PAGE showing ~ 80% purity. Lane M, Protein marker; Lane 1, purified rhBMP2 dimer.
